# Can caregivers report their care recipients’ post-stroke hospitalizations and outpatient visits accurately? Findings of an Asian prospective stroke cohort

**DOI:** 10.1186/s12913-018-3634-4

**Published:** 2018-10-25

**Authors:** Shilpa Tyagi, Gerald Choon-Huat Koh, Nan Luo, Kelvin Bryan Tan, Helen Hoenig, David B. Matchar, Joanne Yoong, Eric A. Finkelstein, Kim En Lee, N. Venketasubramanian, Edward Menon, Kin Ming Chan, Deidre Anne De Silva, Philip Yap, Boon Yeow Tan, Effie Chew, Sherry H. Young, Yee Sien Ng, Tian Ming Tu, Yan Hoon Ang, Keng He Kong, Rajinder Singh, Reshma A. Merchant, Hui Meng Chang, Tseng Tsai Yeo, Chou Ning, Angela Cheong, Yu Li Ng, Chuen Seng Tan

**Affiliations:** 1 Saw Swee Hock School of Public Health, National University of Singapore, 12 Science Drive 2, #10-01, Singapore, 117549 Singapore; 2 Policy Research & Economics Office, Ministry of Health, Singapore, Singapore; 3 Physical Medicine and Rehabilitation Service, Durham VA Medical Centre, Durham, USA; 4 Program in Health Services and Systems Research, Duke-NUS Graduate Medical School, Singapore, Singapore; 5 Lee Kim En Neurology Pte Ltd, Singapore, Singapore; 6 Raffles Neuroscience Centre, Raffles Hospital, Singapore, Singapore; 7 St. Andrew’s Community Hospital, Singapore, Singapore; 8 Mount Alvernia Hospital, Singapore, Singapore; 9 National Neuroscience Institute, Singapore General Hospital campus, Singapore, Singapore; 10 Geriatric Medicine, Khoo Teck Puat Hospital, Singapore, Singapore; 11 St. Luke’s Hospital, Singapore, Singapore; 12 Department of Rehabilitation Medicine, National University Hospital, Singapore, Singapore; 13 Department of Rehabilitation Medicine, Changi General Hospital, Singapore, Singapore; 14 Department of Rehabilitation Medicine, Singapore General Hospital, Singapore, Singapore; 15 Department of Neurology, National Neuroscience Institute, Neurology, Tan Tock Seng Hospital, Singapore, Singapore; 16 Department of Rehabilitation Medicine, Tan Tock Seng Hospital, Singapore, Singapore; 17 Department of Medicine, Yong Loo Lin School of Medicine, National University of Singapore, Singapore, Singapore; 18 Department of Neurosurgery, National University Hospital, Singapore, Singapore

**Keywords:** Healthcare, Validation, Caregiver, Intraclass correlation coefficient, Stroke

## Abstract

**Background:**

Health services research aimed at understanding service use and improving resource allocation often relies on collecting subjectively reported or proxy-reported healthcare service utilization (HSU) data. It is important to know the discrepancies in such self or proxy reports, as they have significant financial and policy implications. In high-dependency populations, such as stroke survivors, with varying levels of cognitive impairment and dysphasia, caregivers are often potential sources of stroke survivors’ HSU information. Most of the work conducted on agreement analysis to date has focused on validating different sources of self-reported data, with few studies exploring the validity of caregiver-reported data. Addressing this gap, our study aimed to quantify the agreement across the caregiver-reported and national claims-based HSU of stroke patients.

**Methods:**

A prospective study comprising multi-ethnic stroke patient and caregiver dyads (*N* = 485) in Singapore was the basis of the current analysis, which used linked national claims records. Caregiver-reported health services data were collected via face-to-face and telephone interviews, and similar health services data were extracted from the national claims records. The main outcome variable was the modified intraclass correlation coefficient (ICC), which provided the level of agreement across both data sources. We further identified the amount of over- or under-reporting by caregivers across different service types.

**Results:**

We observed variations in agreement for different health services, with agreement across caregiver reports and national claims records being the highest for outpatient visits (specialist and primary care), followed by hospitalizations and emergency department visits. Interestingly, caregivers over-reported hospitalizations by approximately 49% and under-reported specialist and primary care visits by approximately 20 to 30%.

**Conclusions:**

The accuracy of the caregiver-reported HSU of stroke patients varies across different service types. Relatively more objective data sources, such as national claims records, should be considered as a first choice for quantifying health care usage before considering caregiver-reported usage. Caregiver-reported outpatient service use was relatively more accurate than inpatient service use over shorter recall periods. Therefore, in situations where objective data sources are limited, caregiver-reported outpatient information can be considered for low volumes of healthcare consumption, using an appropriate correction to account for potential under-reporting.

**Electronic supplementary material:**

The online version of this article (10.1186/s12913-018-3634-4) contains supplementary material, which is available to authorized users.

## Background

Agreement or repeatability is the degree of concordance across two or more data sources (or raters) for the variables studied. Gisev et al. [1] described inter-rater agreement (IRA) as “*the extent to which different raters assign the same precise value for each item being rated*” [1]. Context is important in conducting and reporting agreement studies, as the accuracy of the results is affected by the population characteristics, recall period, type and frequency of utilization and mode of data collection [2].

Stroke survivors are high utilizers of healthcare resources, and studies have tried to quantify the cost of stroke for resource allocation and policy purposes. Such studies often rely on self-reported usage [3–7]. Moreover, stroke as an illness is unique, as it leads to multiple disabilities and varying levels of cognitive impairment, with survivors often depending on a caregiver for different needs, including keeping track of healthcare service utilization. Health services research aimed at understanding service use and improving resource allocation often relies on collecting subjectively reported healthcare service utilization data. With an ageing population, the dependence on caregivers to collect care recipient-related information will significantly increase. Thus, it is important to assess the validity of the healthcare service utilization of stroke patients reported by their caregivers. Most of the work performed on agreement to date has focused on the agreement between different sources of self-reported data, with few exploring the validity of proxy-reported data compared with electronic records [8–11]. Moreover, none of these studies were based on proxy reporters of stroke survivors. The current study will address the abovementioned knowledge gaps.

In addition to a dearth of research on the validity of proxy-reported data for stroke, there is a general methodological issue regarding how data in such studies are treated. Typically, healthcare utilization agreement studies have attempted to either quantify agreement based on traditional measures, such as Cohen’s kappa or the intraclass correlation coefficient (ICC) [8, 10, 12–18], or used a two-step process to extend the analysis to describing the reporting patterns of utilizers [11, 19–26]. Studies often end up dichotomizing or categorizing their healthcare service utilization data (e.g., counts or discrete events), which could potentially result in a loss of power. Addressing this issue, we will consider an approach whereby we will quantify the agreement based on original count data.

## Methods

The primary aim of the current research was to investigate the absolute agreement between caregiver-reported and national claims record-based healthcare service utilization in stroke patients in an Asian setting. The objectives of the study were the following: (i) to report the absolute agreement between the two data sources in the number of healthcare visits post-stroke for inpatient, emergency department (ED), specialist outpatient clinic (SOC) and primary care (PC) services and (ii) to quantify over- or under-reporting by caregivers across different healthcare services utilized by stroke patients, with national claims records taken as the gold standard.

Researchers have previously reported several weaknesses of administrative databases in conducting research, such as limited information on the clinical care delivered, varying accuracy across different data elements, limited information on reasons for utilization or the severity of illness and limited accuracy in identifying clinical diagnoses or procedures [27]. Therefore, few considerations should be considered while assuming an administrative database, such as a claims data record, as a gold standard in research. First, whether the purpose of the research aligns with the purpose of maintaining the administrative database should be considered. The national claims record in Singapore maintains an island-wide database of acute and outpatient healthcare service utilization and associated expenditures. Current research is focused on studying the reporting pattern for healthcare service utilization, requiring us to extract utilization information from the national claims record. Because the information extracted constitutes the primary data fields captured in the national claims record, we are better positioned to assume it as a gold standard with respect to the data fields considered. However, if we were to use an administrative database to diagnose a disease condition, we would be relying on the International Classification of Diseases (ICD) codes or procedural coding within the administrative database, which are inherently plagued with accuracy issues arising due to variations in coding at the physician level and the limited specificity of certain diseases. In this scenario, consideration of an administrative database as a gold standard may not be feasible unless validation studies provide evidence of the accuracy of the information captured [28, 29].

Another consideration regarding healthcare service utilization information is the coverage of services under the administrative database. The national claims record covers inpatient healthcare utilization across both the public and private sectors, while the outpatient healthcare coverage is limited mainly to the public sector or government-run SOCs and polyclinics. Therefore, we are relatively more confident in considering the national claims record as a gold standard for inpatient services than for outpatient services. To elaborate on potential implications, we will be more certain about the over- or under-reporting of caregiver-reported inpatient services, whereas we must interpret outpatient services based on our findings. If we encounter over-reporting of outpatient services by caregivers, we will not be able to comment on whether there is true over-reporting or if it is an artefact due to the limited coverage of outpatient services in the national claims record. However, if our findings suggest the under-reporting of outpatient services by caregivers, we will be more certain of the finding, with the magnitude of under-reporting being a conservative estimate of the true value.

We hypothesized the following: (i) the absolute agreement to be higher for inpatient service use and lower for ED, SOC and PC service use; and (ii) the inpatient service utilization to be over-reported and the ED, SOC and PC service utilization to be under-reported. Our hypotheses are based on previously reported findings, whereby salient or more serious events such as hospitalizations are reported with greater accuracy than are non-salient or less serious events such as outpatient visits [2]. Moreover, proxies over-report hospitalizations and tend to under-report outpatient service use [8, 30].

### Singapore stroke study (S3)

Stroke survivors and caregivers were recruited from all five tertiary hospitals in Singapore from December 2010 to September 2013 under the Singapore Stroke Study (S3). Eligibility criteria included the following: Singaporean or permanent residents, older than 40 years old and residing in Singapore for 1 year of follow-up, stroke was clinically confirmed with imaging evidence, recent diagnosis (symptoms occurring no more than 4 weeks prior to seeking care) and the absence of global aphasia. Caregivers were immediate or extended family members or friends, older than 21 years of age, the main people providing care and taking responsibility for the patient and not fully paid for caregiving.

Caregiver-reported stroke patients’ healthcare service utilization (counts of hospitalizations, ED visits, SOC visits and PC visits) in the past 3 months at each interview over 1 year was considered in the current analysis. The 3-month recall period was determined based on the past literature supporting the association of shorter recall periods with a greater accuracy of reporting utilization [2]. Information across both S3 and the national claims record was matched based on the quarterly availability of healthcare usage data, with data availability ranging from one quarter to all four quarters post-index stroke. We summed the utilization reported across all available 3-month periods or quarters for both data sources, which implies that if caregiver X-reported and national claims record-based healthcare service utilization information is available over the first post-stroke quarter (0–3 months) and second quarter (3–6 months), utilization over these two quarters will be summed, and the absolute agreement for caregiver X will be calculated based on this summed utilization. This quarter-based approach was taken because the caregiver-reported information was captured over each quarter with a consistent recall period of 3 months, and it enabled us to maximize the sample size available for the current analysis.

### Analysis

Agreement analysis is based on quantifying how well two or more data sources (or raters) report concordance on a common phenomenon being studied. This phenomenon in the current study is healthcare service utilization, captured as count variables. Our two data sources are caregivers of stroke survivors (proxy reported) and the national claims record. Two of the commonly used agreement measures are Cohen’s kappa for binary or categorical variables and the ICC for continuous variables. This traditional ICC, commonly used to quantify agreement for continuous data [31, 32], is based on analysis of variance (ANOVA) [33]. Expanding on this approach, we introduce the concept of modified ICC, which can be defined as the proportion of between-group variance by total variation (including both between- and within-group variance) and can be used to quantify agreement for both continuous and count data [34, 35].

$$ Modified\  ICC=\frac{between- group\ variance}{between- group\ variance+ within- group\ variance} $$

Here, between-group variance is a measure of heterogeneity in the utilization pattern of stroke survivors and within-group variance is a result of deviation from the absolute agreement between healthcare service utilization information for a stroke survivor from both data sources. In simple terms, if there is high absolute agreement between both caregiver-reported and national claims record-based utilization information, the within-group variance (in the denominator) would be lower, resulting in a higher value of the modified ICC. For more details regarding the calculation of the modified ICC, refer to Additional file 1.

Adopting a generalized linear mixed model (GLMM)-based approach, agreement for healthcare service utilization between caregiver-reported and national claims record data can be quantified as this modified ICC, with applicability extending to count or discrete event variables. The GLMM approach provides flexibility to not only deal with non-Gaussian variables but also enable the incorporation of random and fixed effects into the model [36]. We incorporated a random intercept for each stroke survivor and a fixed term denoting the data source (0 = national claims record; 1 = caregiver report) to obtain between-group variance and within-group variance, respectively (Model 1). We ran four models in total, one for each service studied, assuming a Poisson distribution for the total visits with log-link. The GLMM approach provided a modified ICC on the original scale (i.e., total number of visits) and the latent scale (i.e., log-transformed scale), and we reported both scales for completeness. We used the bootstrap approach to obtain the 95% confidence interval for the modified ICC estimates (both latent and original scales). From Model 1, the exponentiated value of the coefficient for the data source variable, where national claims data was the reference, indicated the over- or under-reporting of healthcare service utilization by the caregiver (i.e., incidence rate ratio). We also reported the traditional ICC for the number of visits by assuming the total number of visits was Gaussian-distributed. R software version 3.3.3 was used in the analysis [37], with the rptR package used to compute the modified ICC [35]. A *P*-value less than 0.05 was set as the threshold for statistical significance.

## Results

A total of 485 stroke patient-caregiver dyads were available for the current analysis after matching across both databases and excluding those dyads with patient deaths occurring within the observation period of 12 months (*n* = 37).

The baseline socio-demographic characteristics of the stroke survivors are provided in Table 1. The majority of the stroke survivors were less than 65 years of age, Chinese, married, religious, and male. Almost all stroke survivors were admitted to a subsidized ward class for index-stroke episodes, with approximately 8% opting for non-subsidized wards. The proportions of patients with mild, moderate and severe stroke, as measured by the National Institutes of Health Stroke Scale (NIHSS), were 58%, 36% and 6%, respectively. Approximately 45% of the patients were moderately to severely dependent, as measured by the Barthel Index at baseline, with approximately 38% having slight or no dependence and 17% having complete dependence. More than half of the stroke patients had no cognitive impairment at baseline. The average age of caregivers at baseline was 47 years. More than half of them were spousal caregivers, followed by adult-child, siblings and others (distant relatives and friends) as caregivers. The majority were Chinese females, and approximately three-quarters were married. Slightly more than one-third of the caregivers were providing care to multiple care recipients, and approximately three-fourths were co-residing with the stroke patient.

**Table 1:** Socio-demographic and clinical characteristics of utilizers (stroke patients)

Variable		Frequency/Mean	Percent/SD
Age			
	< 65 years	292	60
	≥65 years	193	40
Gender			
	Male	318	66
	Female	167	34
Ethnicity			
	Chinese	326	67
	Non-Chinese	159	33
Religion			
	Religion	440	91
	No Religion	44	9
Marital status			
	Married	347	72
	Single	138	28
Ward class			
	Unsubsidized	39	8
	Subsidized	446	92
Stroke Type			
	Ischaemic	420	87
	Non-ischaemic	62	13
National Institute of Health Stroke Scale (NIHSS)			
	Mild (0–4)	265	58
	Moderately Severe (5–14)	165	36
	Severe (15–24)	25	6
Barthel Index			
	Independence (100)	104	24
	Slight Dependence (91–99)	60	14
	Moderate Dependence (61–90)	128	30
	Severe Dependence (21–60)	65	15
	Total Dependence (0–20)	73	17
Modified Rankin Scale (mRS)			
	No or Slight Disability (0–2)	197	42
	Moderate or Severe Disability (3–5)	277	58
Mini-Mental State Examination (MMSE)	No Cognitive Impairment (24–30)	252	63
	Mild Cognitive Impairment (18–23)	98	24
	Severe Cognitive Impairment (1–17)	53	13
Frontal Assessment Battery (FAB)^a^		14	4
Centre for Epidemiological Studies Depression Scale (CESD)^a^		6	5
Caregiver identity	Spouse	259	54
	Adult-Child	128	26
	Sibling	29	6
	Others	27	6
	None	40	8

^a^The mean and SD were reported for these variables

As shown in Table 2, the caregivers reported outpatient service utilization by stroke patients with greater accuracy than inpatient service utilization. The highest agreement was observed for the SOC service, with a modified ICC value of 0.64 (95% CI: 0.56, 0.69), and this was closely followed by the PC service, with the agreement measured as a modified ICC of 0.61 (95% CI: 0.52, 0.66). Within the inpatient services, the volume of hospitalizations was reported with greater accuracy than was the volume of ED services consumed, with an ICC between a caregiver-reported and claims record-based volume of hospitalizations across both data sources of 0.48 (95% CI: 0.41, 0.55). The lowest agreement was observed for ED services, with a modified ICC value of 0.39 (95% CI: 0.08, 0.49).

**Table 2:** Summary statistics and agreement estimates for healthcare usage by stroke patients over 1 year post-stroke

	Caregiver-reported visits Median (25th – 75th)	Objective administrative visits Median (25th – 75th)	ICC^a^ Estimate (95% CI)	Modified ICC, original scale^b^ Estimate (95% CI)	Modified ICC, latent scale^b^ Estimate (95% CI)	Over- or under-reporting effect IRR (95% CI)
Hospitalization Mean (SD)	0 (0–1) 0.55 (0.89)	0 (0–0) 0.38 (0.93)	0.48 (0.41, 0.55)	0.55 (0.35, 0.63)	0.54 (0.42, 0.61)	1.49 (1.22, 1.82)
ED visits Mean (SD)	0 (0–0) 0.11 (0.42)	0 (0–1) 0.53 (1.38)	0.10 (0.01, 0.19)	0.10 (0.00, 0.11)	0.39 (0.08, 0.49)	0.19 (0.13, 0.28)
SOC visits Mean (SD)	2 (0–4) 2.80 (3.37)	3 (1–5) 3.87 (4.31)	0.51 (0.44, 0.57)	0.59 (0.51, 0.67)	0.64 (0.56, 0.69)	0.71 (0.65, 0.78)
Primary care visits Mean (SD)	1 (0–2) 1.50 (1.96)	1 (0–3) 1.83 (2.14)	0.47 (0.40, 0.53)	0.60 (0.49, 0.69)	0.61 (0.52, 0.66)	0.81 (0.72, 0.91)

^a^ICC assumes the healthcare usage has a Gaussian distribution

^b^Modified ICC assumes the healthcare usage has a Poisson distribution

All the services utilized by stroke patients were under-reported by their caregivers, except for the number of hospitalizations, which was over-reported. Caregivers reported 49% more hospitalizations than those found in the national claims record (95% CI: 1.22, 1.82). The magnitude of under-reporting by caregivers, compared with utilization in the national claims record, varied across different services, with under-reporting ranging from 72 to 87%, 23 to 35% and 9 to 28% for ED, SOC and PC services, respectively (Table 2). We conducted additional sensitivity analyses to determine whether the reported results changed with the addition of relevant covariates. In general, our adjusted analysis results were similar to those reported here after taking into account the variability attributable to the covariates, such as stroke survivors’ socio-demographic, clinical and functional characteristics (refer to Additional file 2: Tables S1, S2 and S3 for details regarding the analyses and results).

## Discussion

Our study demonstrates a discordance between proxy and national claims record reports of health care utilization for stroke patients. Furthermore, we illustrate a novel approach for quantifying agreement across different data sources using the original data (e.g., counts or discrete events), without dichotomizing or categorizing our data. In the past, some studies limited their results to reporting single agreement measures such as kappa or the traditional ICC [8, 10, 12–18], while others opted for a second analysis, reporting factors associated with over- or under-reporting [11, 19–26]. Often with healthcare data, count variables are binarized to facilitate the use of kappa, compromising on the information available, which potentially results in a loss of power, and our study addressed this gap in the literature.

Previous studies have reported that more salient events, such as hospitalizations, are remembered with greater accuracy than are less salient events, such as outpatient visits [2, 11]. However, our results show caregiver-reported agreement to be higher for outpatient (SOC and PC) services compared with that for inpatient services. A possible explanation could be that past literature mainly focused on self-reported estimates, while our study is among the few to analyse caregiver-reported usage and, to the best of our knowledge, the first to do so in stroke populations. Another possible explanation could be the shorter recall period in our study, during which caregivers reported more frequent events with greater accuracy than did less frequent events. In the past, a shorter recall period was recommended to yield a more accurate reporting of frequent healthcare events [2]. Moreover, agreement estimates for salient events are higher for studies involving a longer recall period [11, 30, 38] than a shorter recall period [8, 17]. Another possibility could be that the construct of saliency may be operationalized differently for caregivers, with the frequency of healthcare events having greater significance than the saliency of the type of healthcare event. Similar findings were reported by a study involving proxy-reported healthcare use, with agreement for outpatient service use being higher than that for inpatient service use, with a Lin’s concordance correlation coefficient of 0.67 and 0.55, respectively [8]. Compared with the traditional ICC, higher estimates of the modified ICC were observed for all healthcare services. Therefore, treating count data as continuous may lead to the underestimation of the magnitude of agreement by approximately 15%, 16% and 28% for inpatient, SOC and PC services, respectively.

Assuming the national claims record as a reference, we observed the over-reporting of inpatient service use and under-reporting of outpatient service use by stroke patients according to their caregivers, which is in accordance with previous studies [8, 15, 30]. For example, for every 10 hospitalizations in the national claims database, caregivers reported 15 hospitalizations, whereas for every 10 PC visits, caregivers reported 8 visits. There are potential implications of these inaccuracies in reporting, whereby using caregiver-reported hospitalizations could inflate the usage by 50% and lead to the overestimation of usage and economic burden. This result is important because hospitalizations constitute a major proportion of total healthcare costs. Similarly, the use of caregiver-reported PC usage may yield conservative estimates, with a 20% lower usage; this may affect decision-making in terms of the allocation of resources to outpatient settings or evaluation of interventions targeted at improving outpatient service use post-stroke. Based on current findings, as much as possible, the use of administrative data sources (e.g., national claims record) for healthcare service utilization information is suggested. Depending on the availability and feasibility of acquiring administrative data, caregiver-reported estimates of outpatient service use can be considered for low volumes of consumption, taking into consideration 20 to 30% under-reporting by inflating the caregiver-reported utilization appropriately. When these caregiver-reported estimates of outpatient service usage are independent variables in the regression analysis, measurement error models can be used to avoid biased estimates. It is important to note that the decision to choose one of the data sources, proxy-reported or administrative databases (including national claims records), should not only be based on the accuracy of information but also consider factors such as feasibility, timeliness, availability, cost of acquisition, and comprehensiveness of the data fields collected.

Our study has some limitations. We assumed the national claims record to be the gold standard source of healthcare service utilization data, which may not be the case. Although inpatient service utilization in claims records includes both public and private sectors, PC service utilization may be limited to the public sector. However, the healthcare system in Singapore is based on a heavily subsidized public healthcare sector to enable affordability, which in turn incentivizes consumers to utilize healthcare resources in the public sector. Moreover, our findings of the under-reporting of PC usage are a conservative estimate of the actual under-reporting by the proxies, considering the limited coverage of PC in the national claims record. Because agreement estimates are context-dependent and vary across different populations, the generalizability of our results will be limited to the population of stroke survivors.

Our study has the following strengths. First, we adopted a modelling approach to calculate absolute agreement while accommodating for non-Gaussian healthcare data. Moreover, adopting this methodology allowed us to study both agreement measures and reporting patterns (over- vs. under-reporting) within a single analysis rather than calculating them separately, as has been done in the past [11, 19–26, 39]. Second, to the best of our knowledge, we are among the first to report the agreement of caregivers or proxy-reported healthcare utilization with the national claims record (an objective data source). Recently, a few authors reported agreement for proxy-reported resource utilization [8–11]. However, none of these studies focused on stroke survivor populations, who are relatively high utilizers. The higher relevance of our study also lies in the fact that post-stroke survivors have speech impairment, cognitive impairment, and physical disabilities of varying magnitudes, resulting in a high reliance on caregivers to report the healthcare resource use of their care recipient. Moreover, with a rapidly ageing population globally, the relevance of caregiver-reported resource utilization will increase. Thus, it is crucial to determine the accuracy of their reporting patterns.

## Conclusion

In conclusion, we described the agreement between caregiver-reported and national claims record-based healthcare utilization of stroke survivors by utilizing a model-based approach and reporting the modified ICC. Caregiver-reported outpatient service use is relatively more accurate than inpatient service use over shorter recall periods based on a comparison of modified ICC estimates. Therefore, objective data sources (such as a national claims record) should be considered for quantifying healthcare usage. Depending on the availability and feasibility of acquiring such administrative data, caregiver-reported estimates of outpatient service usage can be considered for low volumes of consumption. Furthermore, the modified ICC can be used for both Gaussian and Poisson data, making the modified ICC a universal agreement measure for continuous and count variables. Future research initiatives should expand the current analysis to study the agreement between caregiver-reported healthcare costs and explore adjusted agreement analysis with appropriate covariates.

## Additional files

Additional file 1: Modified ICC calculation from generalized linear mixed-effects models.pdf. (PDF 99 kb)

Additional file 2: Supplementary tables of adjusted analysis.pdf. (PDF 113 kb)

## Abbreviations

ANOVA: Analysis of variance
ED: Emergency department
GLMM: Generalized linear mixed model
ICC: Intraclass correlation coefficient
IRA: Inter-rater agreement
NIHSS: National Institute of Health Stroke Scale
PC: Primary care
S3: Singapore Stroke Study
SOC: Specialist outpatient clinic
