# Supplementary material for: Can caregivers report their care recipients’ post-stroke hospitalizations and outpatient visits accurately? Findings of an Asian prospective stroke cohort
Source: BMC Health Serv Res. 2018 Oct 25;18:817. doi: 10.1186/s12913-018-3634-4 (PMC6203286; doi:10.1186/s12913-018-3634-4)
Supplement: Supplementary file 1 — Modified ICC calculation from generalized linear mixed-effects models.pdf. (PDF 99 kb) [file 12913_2018_3634_MOESM1_ESM.pdf]

Modified ICC calculation from generalized linear mixed-effects models (GLMMs) with Poisson error structure and log-link.

GLMM structure	$Y_{ij} \sim Poisson\ (\mu_{ij})$<br>$\mu_{ij} = \exp(\beta_0 + \ \alpha_i + \ e_{ij}\ )$<br>$\alpha_i \sim Gaussian\ (0, \sigma_\alpha^2\ )$<br>$e_{ij} \sim Gaussian\ (0, \sigma_e^2\ )$<br><br>where Y_{ij} is the observed count for the *i*th individual at the *j*th occasion, μ_{ij} is the underlying (latent) mean for the *i*th individual at the *j*th occasion, β_0 is the intercept on the link scale, α_i is the individual (random) effect term with a between-individual variance σ_α^2 and e_{ij} is the additive over-dispersion (residual) term on the log-normal scale with a variance of σ_e^2.
Residual variance	$\sigma_\varepsilon^2 = \ \omega + \ln(1/_{\exp(\beta_0)} + 1)$<br><br>where corresponding symbols are same as above with ω being the dispersion parameter for the model.
Modified ICC on latent scale	$ICC_{\text{lnormA}} = \frac{\sigma_\alpha^2}{\sigma_\alpha^2 + \sigma_e^2 + \ln(1/_{\exp(\beta_0)+1})}$<br><br>where ICC_{lnormA} is the log-normal scale ICC for count data with additive over-dispersion.
Modified ICC on original scale	$ICC_{countA} = \frac{E[Yij]_A \cdot (\exp(\sigma_\alpha^2) - 1)}{E[Yij]_A \cdot (\exp(\sigma_\alpha^2 + \sigma_e^2) - 1) + 1}$<br><br>where ICC_{countA} is the count-scale ICC estimated from a Poisson model with additive over-dispersion, $E[Y_{ij}]_{\text{A}}$ is the expectation for Y_{ij}.<br><br>$E[Y_{ij}]_{\text{A}} = \exp(\beta_0 + \frac{\sigma_\alpha^2 + \sigma_e^2}{2})$

Adapted from work by Nakagawa et al [1]

Reference

1. Nakagawa S, Schielzeth H. Repeatability for Gaussian and non-Gaussian data: a practical guide for biologists. *Biological Reviews.* 2010;85(4):935-956.
