# Supplementary material for: Can caregivers report their care recipients’ post-stroke hospitalizations and outpatient visits accurately? Findings of an Asian prospective stroke cohort
Source: BMC Health Serv Res. 2018 Oct 25;18:817. doi: 10.1186/s12913-018-3634-4 (PMC6203286; doi:10.1186/s12913-018-3634-4)
Supplement: Supplementary file 2 — Supplementary tables of adjusted analysis.pdf. (PDF 113 kb) [file 12913_2018_3634_MOESM2_ESM.pdf]

SENSITIVITY ANALYSIS

To explore whether accuracy of agreement estimates varied with stroke survivor characteristics, we adjusted for stroke survivor baseline covariates across models of different healthcare service utilization in the following manner: Model 1 was the base model in the main manuscript with a random intercept for each participant and data source variable incorporated as fixed effect term. Model 2 was adjusted for quarterly availability of data (with a categorical variable capturing data availability across both data sources, ranging from one quarter to all four quarters). Model 3 was adjusted for socio-demographic characteristics of stroke survivor, age (0 = less than 65 years; 1 = 65 years and above), gender (0 = male; 1 = female) and ethnicity (0= non-Chinese; 1=Chinese). Model 4 estimate was adjusted for stroke survivors' clinical and functional characteristics, stroke type (0 = ischemic; 1 = non-ischemic) and functional status measured on Modified Rankin Scale (0 = no or slight disability (0-2), 1 = moderate or severe disability (3-5)). Finally, to adjust for caregiver covariate, we introduced patient reported caregiver identity (a categorical variable with possible values being spousal, sibling, adult-child and others (distant relatives and friends)) at baseline in Model 5. All covariates were introduced as fixed effects across all models. **Supplement Table 1** contains estimates of unadjusted and adjusted ICC on latent scale, **Supplement Table 2** contains estimates of unadjusted and adjusted ICC on original scale and **Supplement Table 3** provides estimates of unadjusted and adjusted IRR for under- or over-reporting by caregivers.

Supplement Table 1. Adjusted agreement estimates (modified ICC on latent scale) for healthcare utilization by stroke patients

	Modified ICC, latent scale† Estimate (95%CI)	Adjusted Modified ICC, latent scale† Estimate (95%CI)			
	Model 1.	Model 2.	Model 3.	Model 4.	Model 5.
Hospitalization	0.54 (0.42, 0.61)	0.52 (0.39, 0.61)	0.55 (0.37, 0.64)	0.52 (0.37, 0.60)	0.54 (0.35, 0.63)
ED visits	0.39 (0.08, 0.49)	0.43 (0.12, 0.51)	#	0.42 (0.08, 0.45)	0.45 (0.09, 0.58)
SOC visits	0.64 (0.56, 0.69)	0.52 (0.44, 0.60)	0.65 (0.59, 0.70)	0.65 (0.57, 0.70)	0.64 (0.54, 0.70)
Primary care visits	0.61 (0.52, 0.66)	0.57 (0.49, 0.62)	0.61 (0.47, 0.64)	0.58 (0.50, 0.64)	0.61 (0.53, 0.67)

†: Modified ICC assumes the healthcare usage has a Poisson distribution.

Model 1 is the base model with a random intercept for each participant and a fixed term for the different data sources; Model 2 is adjusted for quarterly availability of data; Model 3 is adjusted for stroke survivor's socio-demographic characteristics (age, gender and ethnicity); Model 4 is adjusted for stroke survivor's clinical and functional characteristics (stroke type and functional status) and Model 5 is adjusted for caregiver identity.

#: Model had convergence issues.

Supplement Table 2. Adjusted agreement estimates (modified ICC on original scale) for healthcare usage by stroke patients

	Modified ICC, original scale† Estimate (95%CI)	Adjusted Modified ICC, original scale† Estimate (95%CI)			
	Model 1.	Model 2.	Model 3.	Model 4.	Model 5.
Hospitalization	0.55 (0.35, 0.63)	0.53 (0.34, 0.69)	0.58 (0.31, 0.73)	0.53 (0.31, 0.66)	0.56 (0.29, 0.72)
ED visits	0.10 (0.00, 0.11)	0.33 (0.04, 0.45)	#	0.31 (0.02, 0.35)	0.35 (0.02, 0.60)
SOC visits	0.59 (0.51, 0.67)	0.51 (0.41, 0.59)	0.64 (0.56, 0.69)	0.64 (0.56, 0.71)	0.62 (0.51, 0.71)
Primary care visits	0.60 (0.49, 0.69)	0.57 (0.47, 0.64)	0.62 (0.44, 0.66)	0.59 (0.48, 0.67)	0.62 (0.50, 0.69)

†: Modified ICC assumes the healthcare usage has a Poisson distribution.

Model 1 is the base model with a random intercept for each participant and a fixed term for the different data sources; Model 2 is adjusted for quarterly availability of data; Model 3 is adjusted for stroke survivor's socio-demographic characteristics (age, gender and ethnicity); Model 4 is adjusted for stroke survivor's clinical and functional characteristics (stroke type and functional status) and Model 5 is adjusted for caregiver identity.

#: Model had convergence issues.

Supplement Table 3. Adjusted estimate of over- or under-reporting effect by caregivers for healthcare usage by stroke patients

	Over- or under-reporting effect IRR (95%CI)	Over- or under-reporting effect Adjusted IRR (95%CI)			
	Model 1.	Model 2.	Model 3.	Model 4.	Model 5.
Hospitalization	1.49 (1.22, 1.82)	1.48 (1.22, 1.81)	1.49 (1.22, 1.81)	1.48 (1.22, 1.81)	1.51 (1.25, 1.83)
ED visits	0.19 (0.13, 0.28)	0.19 (0.13, 0.28)	#	0.19 (0.14, 0.28)	0.20 (0.14, 0.29)
SOC visits	0.71 (0.65, 0.78)	0.71 (0.65, 0.78)	0.71 (0.65, 0.78)	0.71 (0.65, 0.78)	0.71 (0.64, 0.78)
Primary care visits	0.81 (0.72, 0.91)	0.81 (0.72, 0.91)	0.81 (0.72, 0.91)	0.81 (0.72, 0.91)	0.80 (0.71, 0.90)

†: Modified ICC assumes the healthcare usage has a Poisson distribution.

Model 1 is the base model with a random intercept for each participant and a fixed term for the different data sources; Model 2 is adjusted for quarterly availability of data; Model 3 is adjusted for stroke survivor's socio-demographic characteristics (age, gender and ethnicity); Model 4 is adjusted for stroke survivor's clinical and functional characteristics (stroke type and functional status) and Model 5 is adjusted for caregiver identity.

#: Model had convergence issues.
